# MCI is underdiagnosed among Medicare beneficiaries in the United States

**DOI:** 10.1002/alz70860_099946

**Published:** 2025-12-23

**Authors:** Elyse Couch, Munachimso Ugoh, Lauren Thomas, Emmanuelle Belanger

**Affiliations:** ^1^ Brown University, Providence, RI, USA

## Abstract

**Background:**

Mild cognitive impairment (MCI) is a key risk factor for future dementia. Although MCI affects more people than dementia, little is known about MCI diagnosis rates. The aim of this study was to determine the proportion of people with MCI who received a formal diagnosis and identify patient characteristics associated with an MCI diagnosis in a nationally representative sample of Medicare beneficiaries in the United States.

**Method:**

This study is a retrospective cohort study data from the National Health and Aging Trends Study (NHATS). NHATS is an annual nationally representative survey of Medicare beneficiaries and includes measures of cognition. We included beneficiaries enrolled in the 2015 round of NHATS, who developed symptoms of cognitive impairment consistent with MCI according to a validated algorithm of NHATS measures of cognition, and were continuously enrolled in Medicare fee‐for‐service. Beneficiaries were followed up until 2022. Beneficiaries with diagnosed MCI were identified in Medicare claims data using ICD‐9 and ICD‐10 codes for MCI. Univariate and multivariate logistic regressions were used to identify patient characteristics associated with diagnosed MCI. Characteristics of interest included age, gender, race and ethnicity, marital status, education, number of children, living alone, visiting the doctor alone, number of co‐morbid conditions, depression, and anxiety. Sample weights were applied to all analyses to generate nationally representative estimates.

**Result:**

Of 2.7 million continuously enrolled Medicare beneficiaries with symptoms of cognitive impairment consistent with MCI, only 10.6% had a diagnosis of MCI recorded in claims. Univariate analyses found the odds of diagnosis were lower among younger beneficiaries, higher among women, and lower among beneficiaries who attended doctor visits alone. Multivariate analyses did not find any characteristics significantly associated with diagnosed MCI however, statistical power may have been limited by the small number of beneficiaries with an MCI diagnosis. A sensitivity analysis limiting to significant predictors from the univariate analysis confirmed the association between attending doctor visits alone and undiagnosed MCI.

**Conclusion:**

The findings of this study suggest that MCI is significantly underdiagnosed, particularly among Medicare beneficiaries who attend doctor visits alone. Future research and policy initiatives are needed to increase MCI diagnosis rates.

